# Raumbezogene Gesundheitsdaten und wissenschaftliche Standards zu deren Analyse

**DOI:** 10.1007/s00103-025-04121-6

**Published:** 2025-08-27

**Authors:** Enno Swart, Jobst Augustin, Daniela Koller, Sebastian Völker

**Affiliations:** 1 Medizinische Fakultät, Institut für Sozialmedizin und Gesundheitssystemforschung (ISMG), Otto-von-Guericke-Universität Magdeburg, Leipziger Str. 44, 39120 Magdeburg, Deutschland; 2 Institut für Versorgungsforschung in der Dermatologie und bei Pflegeberufen (IVDP), Universitätsklinikum Hamburg-Eppendorf (UKE), Hamburg, Deutschland; 3 Medizinische Fakultät, Institut für Medizinische Informationsverarbeitung Biometrie und Epidemiologie, LMU München, München, Deutschland; 4 Fachgebiet Gesundheit, IU Internationale Hochschule Erfurt, Erfurt, Deutschland

**Keywords:** Wissenschaftlicher Standard, Gute Praxis, Kleinräumige Analyse, Gesundheitsgeografie, Raumbezogene Daten, Scientific standard, Good practice, Small area analysis, Health geography, Spatial-related data

## Abstract

Raumbezogene Analysen gesundheitsbezogener Daten erfordern eine fundierte Kenntnis raumbezogener Daten(typen) und spezifischer Methoden. Die Auswahl und angemessene Nutzung geeigneter Daten sowie methodischer Verfahren richtet sich nach den inhaltlichen und methodischen Fragestellungen sowie den Zielsetzungen der zugrunde liegenden wissenschaftlichen Studie. Bei der konkreten Planung, Vorbereitung, Durchführung, Datenanalyse und Ergebnisinterpretation wissenschaftlicher Untersuchungen helfen allgemeine und spezifische wissenschaftliche Standards, die als „Gute Praktiken“ (Best Practices) bekannt sind. Der Beitrag definiert und charakterisiert zunächst raumbezogene Daten und beleuchtet typische Fragestellungen sowie methodische Herausforderungen. Eine Übersicht über Gute Praktiken in diesem Bereich wird gegeben. Für raumbezogene Analysen bieten sich die Gute Epidemiologische Praxis, die Gute Kartographische Praxis im Gesundheitswesen und die Gute Praxis Erreichbarkeitsanalysen an, die in diesem Beitrag vorgestellt werden. Für Wissenschaftler:innen bieten sie einen hilfreichen Orientierungsrahmen, von dem allerdings begründet abgewichen werden kann.

## Abstract (en)

Spatial analyses of health-related data require a sound knowledge of spatial data and specific methods. The selection and appropriate use of suitable data and methodological procedures depends on the content and methodological questions as well as the objectives of the underlying scientific study. General and specific scientific standards known as “best practices” help with the concrete planning, preparation, implementation, data analysis, and interpretation of results of scientific studies. This article first defines and characterizes spatial data and highlights typical questions and methodological challenges. An overview of best practices in this area is provided. Good epidemiological practice, good cartographic practice in healthcare, and good accessibility analysis practices, which are presented in this article, are suitable for spatial analyses. They offer scientists a helpful framework for orientation, from which, however, justified deviations are possible.

## Einleitung

Raumbezogene Analysen gesundheitsbezogener Daten haben sich seit Mitte der 1980er-Jahre unter dem Begriff „Small Area Analysis“ etabliert. Wennberg und seine Kolleg:innen zeigten in ihren Untersuchungen, dass Variabilität in der Gesundheitsversorgung eher die Regel als die Ausnahme darstellt. Als Ursachen für diese räumlichen Unterschiede wurden unter anderem Unterschiede in den Angebotsstrukturen, regionale Ausprägungen ärztlicher Versorgungsmuster (Physician Practice Style) und nachfragebezogene Faktoren identifiziert [1–3].

Diese Arbeiten lösten eine zunehmende Zahl vergleichbarer Arbeiten in angelsächsischen und skandinavischen Ländern, später, etwa ab den 2000er-Jahren, auch in Deutschland aus. Zu nennen sind hier etwa Analysen von Abrechnungsdaten der gesetzlichen Krankenkassen (GKV) [4, 5] oder des Zentralinstituts für die kassenärztliche Versorgung („Versorgungsatlas“; [6]), parallel zur Etablierung der Versorgungsforschung in Deutschland.

Derartige Studien im Bereich der Versorgungsepidemiologie und -forschung bedienen sich zwar spezifischer Methoden und Instrumente, fußen aber auf den basalen Verfahren ihrer Grundlagenfächer wie Epidemiologie, Biostatistik, empirische Sozialforschung und Geografie. Entsprechend gelten bei Planung und Vorbereitung, Durchführung, Analyse und Interpretation raumbezogener Forschungsarbeiten die wissenschaftlichen Standards dieser Disziplinen, die allen Forschenden in diesem Feld bekannt sein und reflektiert angewendet werden sollten.

In dem vorliegende Übersichtsbeitrag werden, auch als Grundlage für die weiteren Artikel in diesem Themenheft, zunächst raumbezogene Daten definiert und charakterisiert, die üblicherweise in regionalen Gesundheitsstudien Anwendung finden. Aus den Daten ergeben sich typische methodische und inhaltliche Fragestellungen, für die anschließend wissenschaftliche Standards in Form „Guter Praktiken“ (Best Practices) vorgestellt werden. Diese sollen Forschenden als Handlungsrahmen für Planung und Durchführung raumbezogener Versorgungsstudien dienen. Zuerst wird die *Gute Epidemiologische Praxis (GEP)* mit ihrer spezifischen Ergänzung *Gute Praxis Sekundärdatenanalyse (GPS) *skizziert, gefolgt von Standards für raumbezogene Analysen, der *Guten Kartographischen Praxis im Gesundheitswesen (GKPiG)* und der *Guten Praxis Erreichbarkeitsanalysen (GPEG)*. Der Beitrag endet mit einem Fazit zur konkreten Nutzung dieser wissenschaftlichen Standards im Kontext raumbezogener Analysen.

## Raumbezogene Daten im Gesundheitswesen

Raumbezogene Daten sind auch im Gesundheitswesen von Bedeutung. Sie beinhalten Informationen, die sich einer räumlichen Lage (z. B. über eine Adresse) zuordnen lassen. Raumbezogene Daten werden unterschieden in Vektor- und Rasterdaten. Zu den Vektordaten zählen Punkt‑, Linien- und Polygon- bzw. Flächendaten. Punktdaten ermöglichen z. B. die exakte Verortung von Einrichtungen wie Arztpraxen oder Krankenhäuser über ihre Adresse. Dadurch lassen sich beispielsweise Versorgungsschwerpunkte feststellen oder Verteilungen von Leistungserbringern bewerten [7–9]. Liniendaten umfassen Straßennetze und Netze des öffentlichen Personennahverkehrs (ÖPNV), etwa aus freien Geodatenplattformen wie OpenStreetMap, die für Erreichbarkeitsanalysen unerlässlich sind. Durch sie lässt sich beispielsweise ermitteln, wie schnell Patient:innen bestimmte Versorgungsorte erreichen können [10, 11]. Polygon- oder Flächendaten stellen administrative Einheiten (z. B. Landkreise) dar, aber auch Einzugsgebiete oder Risikozonen. In der Gesundheitsforschung dienen diese – etwa bei kleinräumigen Analysen – dazu, räumlich differenzierte Inzidenz- oder Prävalenzraten zu berechnen und regionale Unterschiede in der Versorgung zu visualisieren [12–14].

**Abb. 1:**
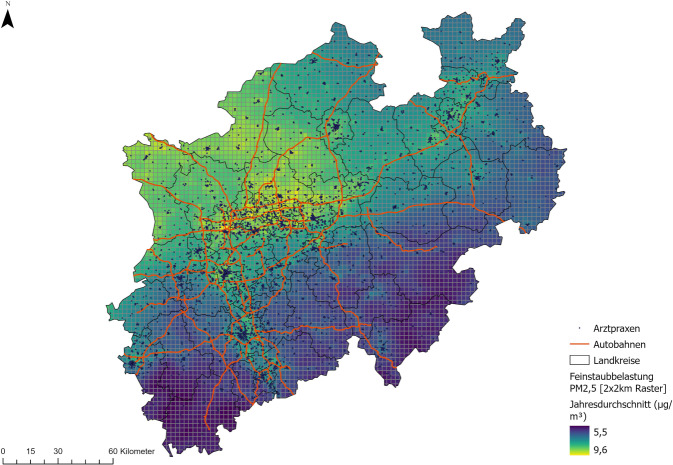
Raumbezogene Datentypen am Beispiel des Bundeslands Nordrhein-Westfalen. Dargestellt sind in Kombination Punkt- (Arztpraxen), Linien- (Autobahnen), Polygon- (Landkreise) und Rasterdaten (Feinstaubbelastung). (Quelle: S. Völker mit Rückgriff auf folgende Datenquellen: Bundesamt für Kartographie und Geodäsie 2025, Umweltbundesamt 2023, OpenStreetMap 2025)

Rasterdaten hingegen stellen Flächen auf einem Gitter dar. Sie bestehen aus gleich großen Bildpunkten (Pixeln) und eignen sich insbesondere zur Darstellung von Bevölkerungsdichten oder Umweltinformationen wie Feinstaubbelastungen, was im Kontext umweltbedingter Gesundheitsrisiken relevant sein kann. Sie erleichtern zudem Kombinationen mit anderen Daten (z. B. Schadstoffbelastung und körperliche Aktivität), die in übergreifenden Public-Health-Studien Anwendung finden [15]. Die verschiedenen Datentypen können auch in einer Darstellung kombiniert Anwendung finden. Ein Beispiel ist in Abb. 1 zu finden. Hier werden administrative Daten (Landkreise) als Polygondaten und Autobahnverläufe als Liniendaten des Bundesamts für Kartographie und Geodäsie, Daten auf Basis der optimalen Interpolation zur Feinstaubbelastung des Umweltbundesamtes als Rasterdaten sowie Daten zu Standorten von Arztpraxen als Punktdaten aus OpenStreetMap in einer Karte dargestellt.

Die Integration dieser unterschiedlichen Datentypen ermöglicht, beispielsweise auch in Kombination mit GKV-Routinedaten, eine detaillierte Beschreibung der Versorgungssituation und fundierte, evidenzbasierte Analysen gesundheitsbezogener Fragestellungen. Sie fördert die Ableitung praxistauglicher Handlungsempfehlungen für Entscheidungs- und Planungsträger, sowohl in der Forschung als auch im öffentlichen Gesundheitsdienst und in der Privatwirtschaft.

## Beispielhafte raumbezogene Fragestellungen und daraus abgeleitete methodische Herausforderungen

Zur Beurteilung, inwieweit wissenschaftliche Standards hilfreich bei Planung, Durchführung und Auswertung kleinräumiger gesundheitsbezogener Studien sind und zur Verbesserung der Qualität derartiger Untersuchungen beitragen können, seien nachfolgend exemplarisch einige typische, inhaltliche Fragestellungen aufgeführt:

- Gibt es räumliche Cluster, die auf eine erhöhte Morbidität und eine überdurchschnittliche Inanspruchnahme hindeuten? Dies könnte auf einen überdurchschnittlichen Versorgungsbedarf oder Qualitätsunterschiede in der Versorgung [16] hinweisen und Anlass für eine Berücksichtigung in Planungsprozessen für den ambulanten oder stationären Versorgungsbereich geben. Ein Beispiel für derartige Analysen stellt der Versorgungsatlas des Zentralinstituts für die kassenärztliche Versorgung dar (www.versorgungsatlas.de; [6]), in dem mehrheitlich das Versorgungsgeschehen auf Ebene von Landkreisen und kreisfreien Städten abbildet wird.
- Ist eine geringere Inanspruchnahme auf eine ungünstigere Erreichbarkeit von Einrichtungen zurückzuführen? Eine derartige Fragestellung ist beispielsweise für Analysen der Inanspruchnahme medizinischer Leistungen in ländlichen Räumen relevant, die beispielsweise eine geringere Ausstattung des ÖPNV-Angebots aufweisen.
- Gibt es Häufungen (Hotspots) einzelner räumlicher Maßzahlen (z. B. Gemeinden mit erhöhter Morbidität)? Dies könnte auf spezifische und in ihrer Wirkung räumlich begrenzte Krankheitsursachen hinweisen. Als Beispiele seien hier Analysen zur gesundheitlichen Belastung durch Kernkraftwerke [17] ebenso wie Regionen mit auffälliger Überinanspruchnahme [18] genannt.
- In welchem Ausmaß korrelieren raumbezogene Indikatoren der Inanspruchnahme mit potenziellen externen Einflussfaktoren, z. B. umweltbezogenen Expositionen (Luftschadstoffe, Lärm) oder angebotsbezogenen Faktoren (Arztdichten, Verfügbarkeit von Großgeräten wie Linksherzkathetermessplätzen)?

Die sich bei der Beantwortung dieser Fragen ergebenden methodischen sowie inhaltlichen Herausforderungen sind z. B.:

- Welche Kriterien definieren eine neutrale und objektive Darstellung von gesundheitsbezogenen Informationen auf Karten und wie können Manipulationen vermieden werden?
- Welches ist das sinnvollste Aggregierungsniveau für die Darstellung von Gesundheitsinformationen, insbesondere unterhalb der Ebene von Landkreisen und kreisfreien Städten?
- Welche Abwägungen müssen vorgenommen werden, wenn mit zunehmend feiner regionaler Auflösung Fallzahlen und damit statistische Power sinken und gleichzeitig das Reidentifizierungsrisiko steigt?
- Wie lässt sich die Diskrepanz zwischen der Notwendigkeit einer hohen räumlichen Auflösung und dem Schutz vor Deanonymisierung in Übereinstimmung mit Datenschutzanforderungen lösen?
- Welche Daten und Methoden müssen bei einer Erreichbarkeitsanalyse berücksichtigt werden?
- Was sind die besten Methoden zur Einbeziehung von Entfernungsinformationen in Karten, um eine realitätsnahe Erreichbarkeit von Gesundheitseinrichtungen darzustellen?
- Welche spezifischen Maßzahlen sind geeignet, um räumliche Korrelationen quantitativ zu messen, und welche Kriterien sollten bei deren Auswahl angewendet werden?
- Wie können moderne geostatistische Methoden eingesetzt werden, um in kleinräumigen Gesundheitsanalysen räumliche Strukturen zu identifizieren?

Diese keinesfalls vollständige Auflistung inhaltlicher Fragestellungen und methodischer Probleme zeigt die thematische Breite raumbezogener Analysen in der Gesundheitsforschung sowie die Herausforderungen ihrer konkreten Umsetzung. Ohne dies aus Platzgründen explizit ausführen zu können, wird implizit deutlich, dass diese raumbezogenen Analysen trotz ihrer Spezifität üblicherweise auf einem epidemiologischen Studiendesign beruhen. Insofern bieten sich für die Entwicklung von Studiendesigns für raumbezogene Analysen sowohl allgemeine epidemiologische wie spezifische wissenschaftliche Standards an.

## Gute Epidemiologische Praktiken

Gesundheitsbezogene Studien mit räumlichem Bezug sind in erster Linie epidemiologische bzw. Versorgungsstudien mit einem ausgeprägten regionalen Bezug, ergänzt um spezifische methodische Ansätze. Insofern gelten für diese Studien entsprechende wissenschaftliche Standards aus diesen Wissenschaftszweigen, die hier kurz vorgestellt werden.

Im Jahr 2000 entwickelten wissenschaftliche Fachgesellschaften in Deutschland auf Empfehlung der Deutschen Forschungsgemeinschaft (DFG) Leitlinien zur „Selbstkontrolle der Wissenschaft“ und definierten gute wissenschaftliche Praktiken für ihre Fachgebiete [19]. Erstmals wurde die Gute Epidemiologische Praxis veröffentlicht [20], die aus 11 Leitlinien besteht, die sich wiederum in mehrere Empfehlungen mit dazu gehörenden Erläuterungen untergliedern (Tab. 1).

**Tab. 1:** Kerninhalte der Empfehlungen der Guten Epidemiologischen Praxis (GEP; [20])

Leitlinie	Titel	Kerninhalte der Empfehlungen
1	Ethik	Notwendigkeit eines Ethikvotums
2	Forschungsfrage	Trennung konfirmatorischer und explorativer Fragestellungen, Formulierung von Hypothesen, Sekundäranalysen
3	Studienplan	Studientyp, Studienbasis, Auswahlverfahren, Verzerrungen, Selektion, Operationalisierung, Studienumfang, Operationshandbuch, Ressourcen
4	Probenbanken	Offenheit gegenüber Studienteilnehmer:innen, Begründung von Zusatzanalysen
5	Qualitätssicherung	Notwendigkeit einer Pilotstudie, Amendments, Schulung des Studienpersonals, Erhebungshandbuch, externe Qualitätssicherung
6	Datenhaltung und -dokumentation	Studiendatenbank, Prüfung Dateneingabe, Rohdatensatz, unabhängige Kodierung, Plausibilitätskontrollen, Auswertedatensatz
7	Auswertung	Auswertungen nach Analyseplan, Begründung von Zwischenauswertungen, Gegenprüfung der Ergebnisse vor Publikationen
8	Datenschutz	Verpflichtung des Studienpersonals, Umgang mit pseudonymisierten und anonymisierten Daten
9	Vertragliche Rahmenbedingungen	Vereinbarungen mit Auftraggeber, Publikationen bei Auftragsforschungen, Vereinbarungen mit den Kooperationspartnern
10	Interpretation	Diskussion der Methoden, Daten und Ergebnisse, externes Review
11	Kommunikation und Public Health	Einbeziehung betroffener Bevölkerungsgruppen, Ableitung von Empfehlungen, Offenlegung der Instrumente, Weiternutzung der wissenschaftlichen Daten

Die GEP will Epidemiolog:innen und in der Epidemiologie beschäftigten Forscher:innen eine Handlungsanleitung für die Planung, Vorbereitung, Durchführung, Analyse und Interpretation epidemiologischer Studien bieten. Wir alle Leitlinien ist sie nicht als strenges Regelwerk zu verstehen, sondern als Orientierung für das eigene Handeln. Dabei kann gut begründet jederzeit von den Empfehlungen der GEP abgewichen werden. Die GEP wurde seit ihrer Veröffentlichung 2‑mal überarbeitet, zuletzt 2019 [21].

Da raumbezogene Gesundheitsstudien häufig mit Sekundärdaten arbeiten, z. B. Abrechnungsdaten gesetzlicher Krankenkassen, Daten amtlicher Statistiken oder umweltbezogene Expositionsdaten, ist ergänzend zur GEP der entsprechende Standard der Sekundärdatenanalyse relevant. Aus der Guten Epidemiologischen Praxis entwickelte sich die *Gute Praxis Sekundärdatenanalyse* (GPS), die 2004 erstmals publiziert wurde und seitdem 2 Revisionen erfuhr, zuletzt 2012 [22]. Sie entstand aus der Erfahrung, dass die Festlegungen der GEP in vielen Punkten den spezifischen Rahmenbedingungen und Voraussetzungen von Sekundärdatenanalysen nicht vollständig gerecht werden. Dies trifft im Besonderen auf die wissenschaftliche Nutzung von Abrechnungsdaten gesetzlicher Krankenkassen zu. Mit der Guten Praxis Sekundärdatenanalyse (GPS) wurde demzufolge ein Standard für die Durchführung von Sekundärdatenanalysen nach wissenschaftlichen Grundsätzen formuliert, der in seiner Struktur und den konkreten Standards der GEP angelehnt und als dessen Ergänzung zu verstehen ist. Sie konkretisiert die in den GEP formulierten allgemeingültigen Qualitätsanforderungen in der Epidemiologie für den Bereich der Sekundärdatenforschung. Zwar stimmen die Leitlinien der GPS mit denen der GEP überein, aber in den Empfehlungen werden die spezifischen Rahmenbedingungen für Sekundärdatenanalysen in Deutschland erkennbar. Dies betrifft besonders die Leitlinien 3 (Studienplan), 6 (Datenhaltung und Datendokumentation) und 8 (Datenschutz).

Zielgruppe der GPS sind alle Personen, die sich unter wissenschaftlicher Perspektive und unter Anwendung wissenschaftlicher Methoden Sekundärdaten, ihrer Analyse und Interpretation zuwenden. Dies schließt auch die Dateneigner ein. Der Wirkbereich dieser Leitlinien bezieht jede Form von Sekundärdaten ein, fokussiert jedoch auf medizinische Sekundärdaten, also typischerweise Routinedaten der gesetzlichen Kranken‑, Renten- und Unfallversicherung (Sozialdaten) oder Daten von (bevölkerungsbezogenen) Krankheitsregistern.

Aktuell wird die GPS von der Arbeitsgruppe Erhebung und Nutzung von Sekundärdaten (AGENS) der Deutschen Gesellschaft für Sozialmedizin und Prävention (DGSMP) und der Deutschen Gesellschaft für Epidemiologie (DGEpi) sowie der Arbeitsgruppe Validierung und Datenlinkage des Deutschen Netzwerks Versorgungsforschung (DNVF) einer erneuten Revision unterzogen.

Auf weitere gute Praktiken im Kontext der Epidemiologie und Versorgungsforschung sei hier nur hingewiesen, so die Gute Praxis Datenlinkage (GPD; [23]) oder die Gute Praxis Gesundheitsberichterstattung [24].

## Gute Kartographische Praxis im Gesundheitswesen (GKPiG)

In gesundheitswissenschaftlichen Studien und der Gesundheitsberichtserstattung werden immer häufiger Karten verwendet. Ursache hierfür ist ein gewachsenes Interesse an regionalen Fragestellungen. Dieser Aspekt ist eingebettet in eine Wende des akademischen Diskurses zum Raum sowie eine bessere Verfügbarkeit von gesundheitlich relevanten Daten mit regionalem Bezug. Mittlerweile können Anwender:innen mittels frei verfügbarer Softwarepakete einfache Daten analysieren und visualisieren.

Bei fehlenden methodischen Kenntnissen für die Erstellung von Karten können Probleme bei der Interpretation kartografischer Darstellungen entstehen. Eine zusätzliche Chance und auch Herausforderung stellt die Interdisziplinarität des Themenbereiches dar, wodurch unterschiedliche methodische Ansätze und fachspezifische Perspektiven einfließen. Handlungsempfehlungen können helfen, die Qualität der Kartenerstellung zu verbessern und die Interpretation zu erleichtern.

Dieser Umstand hat einen Expertenkreis aus Geografie, Kartografie, Epidemiologie und Gesundheitswissenschaften im Jahr 2012 bewogen, in Ergänzung der genannten übergeordneten guten Praktiken eine spezifische „Gute Kartographische Praxis im Gesundheitswesen“ (GKPiG) zu entwickeln [25] die gerade einer ersten Revision unterzogen wurde. Diese dient wie die anderen guten Praktiken als Orientierungshilfe bei der Erstellung und Interpretation von Karten mit gesundheitlichem Bezug nach aktuellen wissenschaftlichen Standards.

Die Gliederung der GKPiG orientiert sich am typischen Entstehungsprozess einer Karte mit gesundheitsbezogenen Informationen (Tab. 2). Vor der eigentlichen Kartenerstellung erfolgt die Planung der Karte, um die Rahmenbedingungen und Anforderungen an die Darstellungsform festzulegen. Diese umfasst die Definition der Zielsetzung, des Mediums und der Zielgruppe. Der weitere Arbeitsprozess gliedert sich in die Arbeitsvorbereitung, die Datenaufbereitung und die Kartenerstellung. Letztere ist wiederum in mehrere Schritte unterteilt ist. Für jede dieser Arbeitsphasen wurden spezifische Empfehlungen zur Umsetzung erarbeitet. Zur besseren Nachvollziehbarkeit der Karte sollten Hinweise zu ihrem Verständnis gegeben werden, um Endnutzer:innen die Interpretation zu erleichtern. Entsprechende Empfehlungen für die Umsetzung wurden ebenfalls formuliert.

**Tab. 2:** Kerninhalte der Empfehlungen der Guten Kartographischen Praxis im Gesundheitswesen (GKPiG; [26, 27])

Kapitel	Titel	Kerninhalte der Empfehlungen
*1*	*Grundlagen*	
1.1	Arbeitsvorbereitung	Art der Darstellung, Zielsetzung der Abbildung, Zielgruppe, Ethik und Datenschutz, Eigenschaften der Daten, Publikationsform, Ausgabeformat der Karten, Ressourcen
1.2	Datenaufbereitung	Datenauswahl und -aufbereitung, Auswahl Kartengrundlage, Wahl raumbezogener Daten, Ausmaß der räumlichen Auflösung, Konsequenzen von Aggregation und Zonierung, Verschneidung von Daten mit unterschiedlichem Raumbezug, Umgang mit administrativen Gebietsveränderungen
*2*	*Kartenerstellung*	
2.1	Räumliche Verteilung der Variablen	Räumliche Muster, Analyse räumlicher Autokorrelation und Identifikation räumlicher Cluster. Übertragung von Punktdaten in Flächendaten
2.2	Kartenproduktion	Thema der Karte, Kartenrandangaben und Legende, Ordnung und Balance im Layout, Kartentypen, mehrschichtige Karten, Kartenprojektion, Signaturen, Flächensignaturen, Klassifizierung, Kartenbeschriftung, Kartenausgabe
2.3	Webbasierte Kartografie	Gesamtkonzept, Besonderheiten der Interaktion, Ressourcen, Barrierefreiheit, Kartenexport
*3*	*Kommunikation und Interpretation*	Interpretation von Karten, Transparenz der Methoden

In der Kurzfassung der GKPiG [26] sind die einzelnen Empfehlungen lediglich kurz erläutert. In der Langfassung [27] finden sich ergänzend zahlreiche Beispiele mit Interpretationshinweisen. Damit wird deren Anschaulichkeit erhöht und die Anwendung dieser guten Praxis gefördert.

Zur Sicherung der Qualität von Kartendarstellungen im Gesundheitswesen sollen die vorliegenden Empfehlungen als Orientierung dienen. Angesichts der vielfältigen Gestaltungsmöglichkeiten von Karten, der damit verbundenen Gefahr von Fehlinterpretation und der besonderen Bedeutung von Karten als Kommunikationsmittel tragen alle Kartenersteller:innen eine besondere Verantwortung [26, 27]. In der Leitlinie werden zudem Beispiele für Software und Pakete genannt, die sowohl für kartografische Darstellungen als auch für räumlich-statistische Verfahren hilfreich sein können.

Adressat:innen dieser Empfehlungen sind alle im Gesundheitswesen tätigen Personen, insbesondere aus den Disziplinen Medizin, Epidemiologie, Versorgungsforschung, Public Health, Gesundheitsökonomie und dem Öffentlichen Gesundheitsdienst, sowie Journalist:innen, Publizist:innen und Infografiker:innen, welche gesundheitliche Sachverhalte kartografisch verarbeiten wollen. Die GKPiG kann zwar keine konkreten Hinweise zu inhaltlichen Interpretationen von Karten geben, adressiert im Kapitel 3 jedoch Aspekte, die bei der Interpretation von Karten und der dazugehörenden Berichtserstattung beachtet werden sollten.

Die GKPiG soll eine Orientierungshilfe in Form von Empfehlungen zur Erstellung und Interpretation von Karten mit gesundheitlichem Bezug nach aktuellen wissenschaftlichen Standards bieten. Die kartografische Bearbeitung gesundheitlicher Fragestellungen soll durch die GKPiG nicht eingeengt werden, sondern vielmehr als Unterstützung für nicht kartografisch oder geografisch ausgebildete Akteur:innen verstanden werden.

## Gute Praxis Erreichbarkeitsanalysen im Gesundheitswesen (GPEG)

Die Gewährleistung gleichwertiger Lebensverhältnisse erfordert, dass Einrichtungen der Gesundheitsversorgung unabhängig vom Wohnort erreichbar sind. Aufgrund der zunehmenden Disparitäten zwischen städtischen und ländlichen Regionen sowie politischer Vorgaben zu Erreichbarkeitsparametern (z. B. Fahrzeitminuten, Entfernung in Kilometern) sind Erreichbarkeitsanalysen zu einem zentralen Planungs- und Steuerungsinstrument geworden. Sie helfen, Versorgungslücken zu identifizieren und (gesundheits-)politische Entscheidungen zu begründen.

Erreichbarkeitsanalysen beruhen auf vereinfachenden Annahmen, etwa zu Bevölkerungsbewegungen, Verkehrsbelastungen und Fahrtdauerprofilen, als auch bzgl. der Datenverfügbarkeit. In der Praxis existieren große Unterschiede bzgl. Methodik, räumlicher Maßstäbe und Einsatz digitaler Tools. Dabei verknüpfen Akteur:innen, die oft unterschiedliche Interessen verfolgen, verschiedene Datenquellen, Methoden und Visualisierungen. Daher plädiert die GPEG für eine sorgfältige Dokumentation und Offenlegung aller Schritte, um Vergleiche zwischen unterschiedlichen Studien zu ermöglichen und Ergebnisse valide interpretieren zu können. Ziel der GPEG ist es, vergleichbare, transparente und nachvollziehbare Analysen zu gewährleisten [28].

Die Entwicklung der GPEG erfolgte in einer Initiative aus Geografie, Epidemiologie und Gesundheitswissenschaften. Dabei wurde einerseits auf bereits vorhandene gute Praktiken wie die „Gute Kartographische Praxis im Gesundheitswesen (GKPiG)“ Bezug genommen, andererseits ein eigenständiger Leitfaden für Erreichbarkeitsanalysen erarbeitet, um die Qualität und Vergleichbarkeit von Studien zu erhöhen.

Die Qualität der Erreichbarkeitsanalyse hängt wesentlich von den eingesetzten Daten ab. Hierzu zählen amtliche Raumeinheiten, standortbezogene Sachdaten und routingfähige Straßendaten. Grundsätzlich gilt es, die jeweils aktuellsten Quellen für die Analyse zu verwenden. Zusätzlich sollen möglichst einheitliche Quellen verwendet werden, sodass die verwendeten Daten in einem standardisierten Format vorliegen und den gleichen methodischen Standards folgen. Neben amtlichen Quellen können nichtamtliche Daten ergänzt oder validiert werden (z. B. OpenStreetMap für Straßen- oder Geofachdaten). Die GPEG empfiehlt die Unterscheidung der Start- und Zielpunkte, wobei adressgenaue Standorte medizinischer Leistungserbringung sowie Gebiete mit hoher Bevölkerungsdichte oder anderen relevanten Bevölkerungsmerkmalen herangezogen werden sollten. Entsprechend dem GPEG-Workflow umfasst eine Analyse die Hauptschritte Auswahl der Geodaten, Bestimmung der Standorte von Leistungserbringern, Definition von Start- und Zielpunkten, Verkehrsmittelwahl und Routingparameter sowie Ergebnisdarstellung (Tab. 3).

**Tab. 3:** Kerninhalte der Empfehlungen der Guten Praxis Erreichbarkeitsanalysen im Gesundheitswesen (GPEG; [28])

Grundsatz	Titel	Kerninhalte der Empfehlungen
1	Geodaten	Begründete Auswahl und Aktualität der Datengrundlagen (amtliche und nichtamtliche Raumeinheiten, sachdatenbezogene Verknüpfungen) als Basis für die Qualität der Erreichbarkeitsanalyse
2	Standorte von Leistungserbringern	Erhebung möglichst genauer, validierter Standortdaten (z. B. Krankenhäuser, Arztpraxen) unter Berücksichtigung von Datenschutz, amtlichen und nichtamtlichen Quellen
3	Start- und Zielpunkte	Festlegung passender Bevölkerungsschwerpunkte oder adressgenauer Standorte; Präferenz für Zentroid- bzw. feinräumige Punkte zur realistischen Abbildung der Versorgungsbereiche
4	Verkehrsmittelwahl und Routing	Auswahl des Verkehrsmittels (motorisierter Individualverkehr, MIV; öffentlicher Personennahverkehr, ÖPNV; Rad- oder Fußverkehr) entsprechend der Fragestellung; Nutzung geeigneter routingfähiger Daten und Berücksichtigung aktueller Geschwindigkeits- und Fahrplanprofile
5	Analyseeinstellungen	Sorgfältige Festlegung der Routing-Parameter (z. B. Geschwindigkeitsprofile, Richtungsbeschränkungen) und Transparenz hinsichtlich ihrer Auswirkungen auf das Analyseergebnis
6	Ergebnisdarstellung	Nachvollziehbare und transparente Aufbereitung, z. B. in Form von Text, Tabellen, Karten; Anwendung der „Guten Kartographischen Praxis im Gesundheitswesen“ (GKPiG) zur Qualitätssteigerung und besseren Interpretierbarkeit

Die „Gute Praxis Erreichbarkeitsanalysen im Gesundheitswesen (GPEG)“ leistet einen wertvollen Beitrag zur Methodentransparenz und Qualitätssicherung von Erreichbarkeitsanalysen. Sie reagiert damit auf das wachsende Bedürfnis, medizinische Versorgungseinrichtungen anhand von nachvollziehbaren, standardisierten Kriterien zu bewerten. Ohne eine solche Empfehlung bestünde das Risiko, dass heterogene Methoden, uneinheitliche Datenquellen und unterschiedlichste Interpretationsebenen zu nicht vergleichbaren Ergebnissen führen. Dies könnte Fehlplanungen und Missverständnisse begünstigen, insbesondere in interdisziplinären Teams, die sowohl medizinisch-epidemiologische als auch geografische Fragestellungen bearbeiten. Die GPEG zielt auf die Unterstützung von Wissenschaft, Politik und Praxis. Sie soll helfen, valide und nachvollziehbare Aussagen zur Erreichbarkeit in der Gesundheitsversorgung zu treffen.

## Fazit

Gesundheitsbezogene Analysen raumbezogener Daten im Bereich Epidemiologie, Versorgungsforschung und Public Health bedürfen nicht zwingend und ausschließend eigener Methoden und Datenzugänge. Vielmehr gilt es zunächst, die wissenschaftlichen Standards der Dachdisziplinen zu kennen, sofern sie sich auf empirische Studiendesigns und typische Datenzugänge über Primär- und Sekundärdaten beziehen. Dazu gehören zuallererst die Gute Epidemiologische Praxis, die Gute Praxis Sekundärdatenanalyse und die Gute Praxis Datenlinkage. Darauf aufbauend geben die Gute Kartographische Praxis im Gesundheitswesen und die Gute Praxis Erreichbarkeitsanalysen spezifische Handlungsempfehlungen für raumbezogene Versorgungsanalysen. Perspektivisch werden modernere geostatistische Methoden, wie hierarchische Modelle oder bayesianische Verfahren, ausführlicher in der sich in Arbeit befindlichen „Guten Praxis räumliche Statistik“ aufgegriffen.

Wie alle guten Praktiken sind diese nicht als starres Handlungskorsett, sondern als Richtlinie und Orientierung bei Planung, Vorbereitung, Durchführung, Datenanalyse und Interpretation der Studienergebnisse zu verstehen. Dabei sollen sie Möglichkeiten zum Abweichen von diesen Empfehlungen bieten, dabei aber die beteiligten Wissenschaftler:innen zur Reflexion und zur Begründung ihrer Vorgehensweise und Festlegungen veranlassen.
